# Acrylamide in Baby Foods: A Probabilistic Exposure Assessment

**DOI:** 10.3390/foods10122900

**Published:** 2021-11-23

**Authors:** Francesco Esposito, Agata Nolasco, Francesco Caracciolo, Salvatore Velotto, Paolo Montuori, Raffaele Romano, Tommaso Stasi, Teresa Cirillo

**Affiliations:** 1 Department of Public Health, University of Naples Federico II, Via Sergio Pansini, 5, 80131 Naples, Italy; pmontuor@unina.it; 2 Department of Agricultural Sciences, University of Naples Federico II, Via Università, 100, 80055 Naples, Italy; agata.nolasco@unina.it (A.N.); francesco.caracciolo@unina.it (F.C.); raffaele.romano@unina.it (R.R.); tcirillo@unina.it (T.C.); 3 Department of Promotion of Human Sciences and the Quality of Life, University of Study of Roma San Raffaele, Via di Val Cannuta, 247, 00166 Roma, Italy; salvatore.velotto@uniroma5.it; 4 Department of Science and Technology, Newton Consulting Srl, 80146 Naples, Italy; tommasostasi@newtonconsulting.it

**Keywords:** dietary exposure, acrylamide, biscuits, margin of exposure, weaned infants

## Abstract

Acrylamide (also known as 2-propenamide) (AA) is a toxicant that develops in food during high-temperature cooking, and its occurrence is common in biscuits and baked snacks. AA is known for its in vivo neurotoxic and carcinogenic effects, and it is considered a potential carcinogen for humans. Infants may be exposed to AA as early as during weaning through baked food such as biscuits. This study set out to ascertain the concentration of AA in food products intended for infants to assess the dietary exposure to this food contaminant. AA levels were determined through GC/MS and bromination, and dietary exposure was evaluated by a probabilistic method based on Monte Carlo simulation. The results showed that the probability of a carcinogenic exposure is 94%, 92%, and 87%, respectively, for 6-, 12-, and 18-months infants, suggesting the need to delay the introduction of baked products in the diet of weaned infants. It should be noted, however, that these conclusions were drawn considering the biscuits as the primary source of exposure.

## 1. Introduction

Acrylamide (AA) is a heat-induced toxicant (i.e., a compound that is formed in starchy foods during high-temperature cooking). This process involves reducing sugars and amino acids (asparagine) naturally present in many foods [1,2]. The predominant mechanism in the AA formation in food is due to the Maillard reaction, which, in addition to the formation of aromatic compounds that enhance the taste and smell of freshly cooked food, leads to potentially dangerous compounds [3,4]. The occurrence of AA in food was first identified by researchers from the Swedish National Food Administration [5] and the University of Stockholm by analyzing foods rich in carbohydrates and cooked at temperatures above 120 °C [6,7]. Pioneering studies conducted by Tareke et al. [8] showed how fried feed given to rats led to a considerable increase of the adducts of AA with N-termini of hemoglobin, regularly observed even in people with no known exposure to AA. Assuming, therefore, that baked food could be one of the primary sources of AA in humans and warning about possible carcinogenic risks [9].

The toxicity of AA has been known for a long time thanks to studies conducted on laboratory animals (mainly rodents) which developed genetic mutations and tumors after being subjected to AA orally [10,11]). Precisely for this reason, since 1994, the International Agency for Research on Cancer IARC has classified acrylamide as a “probable human carcinogen” (Group 2A) [12]. A massive amount of other scientific evidence has been collected over the years to confirm the neurotoxic and carcinogenic potential exerted on animals [13,14,15] and latent risks for human health [16,17,18,19]. A recent pathological study conducted on 30 Wistar rats also stated that the intake of a diet containing AA-induced kidney injury, hepatocellular insufficiency, and chronic liver disease, with subsequently primary immunodeficiency and activation of the immune system due to the possible expression of some immunoreaction genes [20]. In 2015, EFSA published a comprehensive risk assessment of AA in food, highlighting the highest levels of dietary exposure to AA in the following food products: fried potato products, coffee, crackers and crusty bread, and soft bread and biscuits, responsible for up to 49%, 34%, 24%, and 23% of average exposure in adults, respectively [21]. Currently, numerous investigations have been carried out on the AA content present in various foods of different categories that are readily available on the market, but the worrying data is that the risks to human health due to the high concentrations in foods are still high [1,2,22,23,24,25,26,27,28,29,30]. It is noteworthy that there is increasing attention worldwide being given to this problem. However, there are still very few scientific works on evaluating the quantity of AA in foods for infants and children, despite this being the age group most exposed to risks concerning their low body weight [21]. The results of the meta-analysis work conducted by Khaneghah et al. [31] are interesting, attesting that acrylamide concentrations in baby food (156.30 µg/kg) exceed the benchmark level set by Regulation (EU) 2017/2158 equal to 40 and 150 µg/kg for baby food and biscuits, respectively [32].

Concerning the baby-food products available in Italian markets, although extensive research has been carried out on the occurrence of AA in heat-processed products, there is little information in the literature, mainly limited to preliminary studies [33]. It is necessary to consider the rapid evolution of food manufacture as well as the processes used to produce certain foods and the attention paid to the quality of raw materials. More recent studies on products sampled in Italy were conducted by Capei et al. [34], who considered AA concentrations in baked products such as biscuits and breakfast cereals, by Branciari et al. [3], who evaluated the dietary exposure of schoolchildren to AA in a selected population represented by children aged from 3 to 13, and by Esposito et al. [23] on the occurrence of AA in bread sweets and other baked products. However, these studies that even show a renewed interest in the occurrence of this food contaminant also in terms of dietary exposure of the Italian population, did not focus on those products specifically intended for infants.

It is clear, hence, that there is a need to find increasingly effective strategies and solutions for the reduction of this contaminant given the importance of these foods and the vulnerability of the exposed population. For this purpose, this investigation aims to estimate the concentration of AA in food products for infants in order to address the lack of data on the probable dietary exposure of the most susceptible age groups.

## 2. Materials and Methods

### 2.1. Food Sampling

A total of 90 samples were collected in various shops and supermarkets in the Campania region (Italy). Samples included ground and whole biscuits, baby cereal meal, sweet and savory snacks, and baby food with plum puree. All samples were intended for weaned infants, particularly from the 4th to the 36th month of life. Solid samples were homogenized to a final particle size of 500 µm before analysis.

### 2.2. Reagents and Equipment

All of the reagents that were used in this research were GC grade and were supplied by Merck KGaA, (Darmstadt, Germany), namely n-hexane, ethyl acetate, and acetonitrile (ACN). The salts and acids used in this study were supplied by Sigma Aldrich (St. Louis, MO, USA) and were analytical grade: potassium bromide, sodium chloride, hydrobromic acid (48% *w*/*w*), and sodium thiosulfate pentahydrate. The standards AA (purity ≥ 99.9%), and the internal standard (IS) AA−^13^C_3_, were purchased from Supelco (Supelco Inc., Bellefonte, PA, USA); preparative C18 (125 Å 55–105 µm) was provided by Waters (Milford, MA, USA) and bromine water (3% *w*/*v*) was purchased from Titolchimica (Pontecchio Polesine, Italy). An Agilent (Model number 7890A) GC-MS system paired with an Agilent (Model number 5975C) selective mass detector (MSD) (Agilent Technologies, Santa Clara, CA, USA); the capillary column was a Restek Rxi^®^-XLB GC (length × ID.: 30 m × 0.25 mm; df: 0.25 µm), supplied by Restek, Bellefonte, PA, USA).

### 2.3. Analytical Standards

A stock standard solution of 1 g/L was made by dissolving 50 mg of AA in ACN to a final volume of 50 mL, whereas a working solution of 1000 µg/L was obtained by suitable dilution of the stock standard. The standards were analyzed in parallel to the samples and brominated, using aliquots of 750, 500, 250, 125, 63 and 31 µL of working solution (corresponding to a range of 62.5–1000 µg/kg in the samples). Similarly, a working solution of 10 mg/L of ^13^C_3_-IS was prepared accordingly.

### 2.4. Analytical Method

The quantification of AA was carried out according to the methods of previous studies [1,35] through a matrix solid-phase dispersion (MSPD) extraction method and bromination of collected aqueous extracts. 0.50 g of finely ground sample (particle size ≈500 µm) was added to 2 g of C18 and mixed in a glass mortar. Subsequently, the mixture was put into a polypropylene syringe, putting a frit at the bottom and onto the top of the mixture. The syringe was placed onto a vacuum manifold, and the sample was firstly defatted with 20 mL of n-hexane at a flow rate of 4 mL/min. The syringe was dried under vacuum for 2 min. Afterward, AA was collected by adding 5 mL of HPLC grade water, holding the flow for 10 min to favor the extraction of the target molecule, repeating this step twice. Finally, the eluate was collected in a glass vial and underwent bromination, according to the procedure described in the following section. Each sample was analyzed in triplicate.

### 2.5. Bromination Procedure

The first step of this phase involved the addition of 1 g of potassium bromide to the eluates, then two drops of HBr (48% *w*/*w*) were added to the solution to adjust the pH to 2–3. Afterward, 2 mL of bromine water (3% *w*/*v*) were added to the extracts and stored at 0 °C for 2 h in the dark. Few drops of sodium thiosulfate 1 M were added to the brominated extracts until the yellow color of the solution disappeared. Finally, 4 g of sodium chloride were added to the extract, and the target molecule was extracted with a 10 mL ethyl acetate:n-hexane mixture (4:1 *v*/*v*), pooling the upper layers. This step was repeated again with 5 mL of the mixture. The pooled layers were dried to 2 mL under a gentle stream of nitrogen at 40 °C, and half a spatula of sodium sulfate was added. The sample was centrifuged at 5000 rpm for 10 min, and then transferred to a graduated glass vial and dried up to 0.5 mL under a stream of nitrogen before GC analysis.

### 2.6. GC/MS Detection

The analysis was carried out through a GC equipment coupled with a MS detector under the following parameters: the carrier gas was helium, and the flow-rate was 1.0 mL/min; the volume of injected sample was 1 µL in pulsed splitless mode; the temperature of injector was 280 °C. As the run started, the column was held at 85 °C for 1 min, after the rate was 15 °C/min until a temperature of 280 °C, holding it for 18 min for a total run-time of 32 min. The transfer line of the GC-MS interface was set at a temperature of 280 °C. The retention time (RT) of the target molecule and the IS was 7.60 min. The MSD operated in electron ionization (EI) mode; the collision energy was 70 eV. The run was performed in selected ion monitoring mode (SIM mode), and the quantifier ion was *m*/*z* 150 (153 for the IS), whereas the qualifier ions were *m*/*z* 106, 108, and 152 for the derivative 2,3-dibromopropionamide (109 and 155 for the IS).

### 2.7. Analytical Quality Assurance and Method Performance

The calibration curve was plotted as the peak area ratio of the brominated derivative to the IS derivative versus concentration. The limits of detection (LOD) and quantification (LOQ) were calculated respectively using the standard deviation (SD) of the response (σ) and the slope of the calibration curve (S) according to the formula: LOD = 3.3 σ/S. The LOQ was calculated as three times the LOD value. LOD and LOQ were respectively equal to 10 ng/g and 30 ng/g. Recovery tests were performed spiking three aliquots (0.5 g in triplicate) of ground biscuits sample and three aliquots of plum puree, with AA standard and IS at the following spiking levels: 125 ng/g + 500 ng/g (IS), 250 ng/g + 500 ng/g (IS), 500 ng/g + 500 ng/g (IS) and 500 ng/g + 500 ng/g (IS). The recoveries varied between 93% to 98%. Eventually, the precision of the method was calculated, evaluating the intra- and inter-day repeatability through the injection of three samples that were already used for the recovery tests, five times a day and for seven consecutive days. The relative standard deviation (RSD) of the intra-day repeatability was below 7%, whereas the RSD of the inter-day repeatability was below 8%.

### 2.8. Monte Carlo Simulations

The probabilistic exposure assessment to acrylamide in baby foods was carried out through Monte Carlo simulations (MCS). MCS is a computational method widely used in probabilistic analysis to obtain distributions of the outcomes: It addresses the uncertainty of the parameters and it is based on several iterations of random sampling. The MCS was conducted in STATA version 16.1 (Stata Corp, College Station, TX, USA). In order to obtain accurate and stable results, the MCS was set to perform 10,000 iterations [36].

### 2.9. Dietary Exposure to Acrylamide

In order to evaluate the exposure of infants to acrylamide from weaning up to 18 months of age, a dietary exposure assessment was performed. It was carried out considering the same body weight either for males or for females since the weight differences in the first 18 months of life is insignificant. Consumption data are based on the comprehensive European Food Consumption Database [21], whereas body weight (BW) was derived from the growth curves of the World Health Organization [37] and were 8.0, 9.4 and 10.9 kg, for 6, 12, and 18 months infants, respectively. Based on the results of this study, the dietary exposure assessment was performed considering the biscuits as the major contributor in terms of AA intake.

The exposure was calculated according to the following formula:

E = (Q × C)/BW(1)

where E is the daily exposure of AA (ng/kg body weight/day); Q is individual food daily consumption of infants within different age groups (6, 12 and 18 months (g/day); C is the concentration of AA in food (ng/g); and BW is individual body weight (kg body weight)

Several sources of uncertainty co-occur in the exposure calculation described in Equation 1, including, in particular, the limits of analytical methods and the huge variability of the individual food daily consumption. To overcome these challenges, rather than using a single-point value of the variable, the exposure distribution will be assessed through Monte Carlo simulations (MCS). MCS is here used for obtaining a probabilistic assessment of the AA risk and considering the concentration of AA in food (C) and the individual food daily consumption of infants (Q) as random variables defined by probability distributions.

The risk characterization was performed through a Margin of Exposure approach. The MOE is defined as the ratio between the Benchmark-Dose Level 10 (BMDL_10_) that is the lower limit of the 95% confidence interval relating to the reference dose for a 10% response:

MOE = BMDL_10_/E (2)

The BMDL_10_ values considered for the neurotoxic risk were 0.43 mg/kg body weight/day, whereas for the carcinogenic risk, two values of 0.17 and 0.31 mg/kg/body weight/day were considered, derived from the evidence to cause Harder gland cancer and mammary gland cancer in mice, respectively [21]. The exposure estimate was carried out on three categories of the population based on age (i.e., children aged 6, 12, and 18 months). More adult populations were not considered as consumption estimates due to limited data that could be biased.

## 3. Results and Discussion

About 80% of the samples showed detectable AA levels with concentrations ranging between <LOD and 109 ng/g. The AA content in biscuits intended for infants varied from 15 to 109 ng/g; in granulated biscuits, the values were lower than those of the other biscuits. Multigrain soluble creams and sweet snacks showed concentrations between <LOD and 15 ng/g, whereas in salty snacks the concentration was <LOQ (Table 1).

The AA values found in biscuits and sweet snacks were lower than those found by other authors that evaluated AA levels in baby food from Poland and Turkey [38,39,40]. Instead, they are in line with the study results by EFSA (EFSA, 2015). However, it is essential to bear in mind that these studies were concluded before the entry into force of the Regulation (EU) 2017/2158—mitigation measures and benchmark levels for the reduction of the presence of acrylamide in food. The concentrations of AA found in multigrain creams are lower than those found by other authors and all below the LOD [21,37,38,39]. The concentrations observed in this investigation are far below those surveyed by Başaran and Aydın, who reported an average AA level of 233 ng/g in baby biscuits sold in Turkey, with a range between 12 and 1270 ng/g [41]. According to Lambert et al., the AA levels that occurred in biscuits (reported as plain dry biscuits) showed an AA level of 102 ng/g, even though just one sample was considered in that case and it was indicated as a typical food (not intended for infants) [42]. Also, according to the same study, two samples of biscuits intended for infants and the cereals to be reconstituted (comparable with the Multigrain meal analyzed in our investigation) showed an upper-bound level respectively equal to 77 and 17 ng/g, and these results corroborate the data of our study, although no multigrain sample analyzed in our study showed detectable levels of AA.

As far as we know, no study surveyed the occurrence of AA in salty snacks and granulated cookies and the low levels detected, and the low consumption rate of this kind of product suggested a negligible contribution in the dietary exposure of AA. Surprisingly, the plum puree-based food showed lower AA values than the literature data [21]. However, there are several possible explanations for this result, considering that the final concentration of AA in plum-based products depends on quite a few factors, such as plum species and time/temperature pairs during the baking process [43,44].

The AA concentrations detected in the samples were lower than the values issued by the EC regulation 2017/2158. However, to assess the dietary exposure to AA, assessing the margin of exposure (MOE) is advisable.

### Dietary Exposure to Acrylamide of Infants

**Figure 1:**
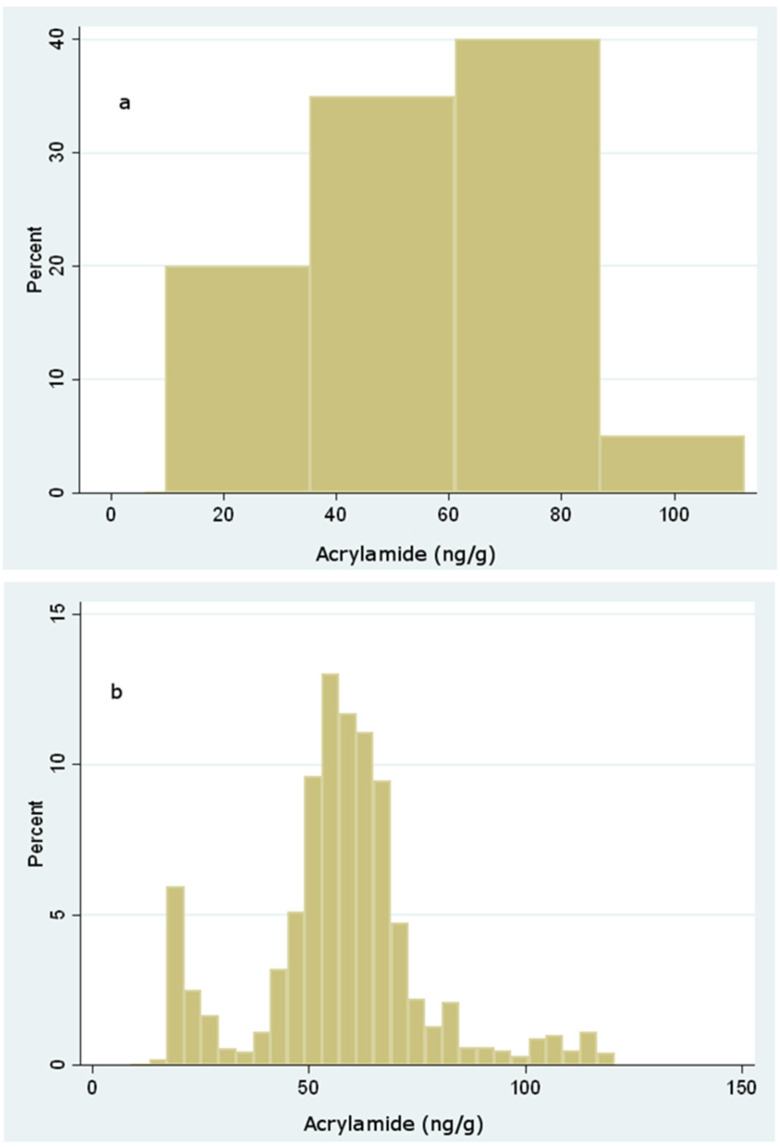
Observed (**a**) and simulated (**b**) distribution of acrylamide levels in whole and ground biscuits.

The probability distribution of C was obtained through bootstrapping with 1000 replicates from the empirical data collected. Moreover, a normally distributed measurement error was added to each observation for simulating the observed uncertainty of the assays (mean = 0; std. dev. = 4). The following graphs show, respectively, the starting empirical data and the simulated distribution of C, including only whole and ground biscuits that actually were considered as the main contributors to dietary exposure (Figure 1).

The individual food daily consumption of infants (Q) is assumed to be log-normally distributed with mean and standard deviation parameter values provided by the Comprehensive European Food Consumption Database by EFSA [21].

The left-censored values were evaluated following a middle-bound approach and were considered as LOQ/2 and LOD/2 for the values occurring below LOQ and LOD, respectively. The margin of exposure (MOE) relating to the risk of neurotoxicity and carcinogenicity in humans was calculated according to Equation (2).

**Figure 2:**
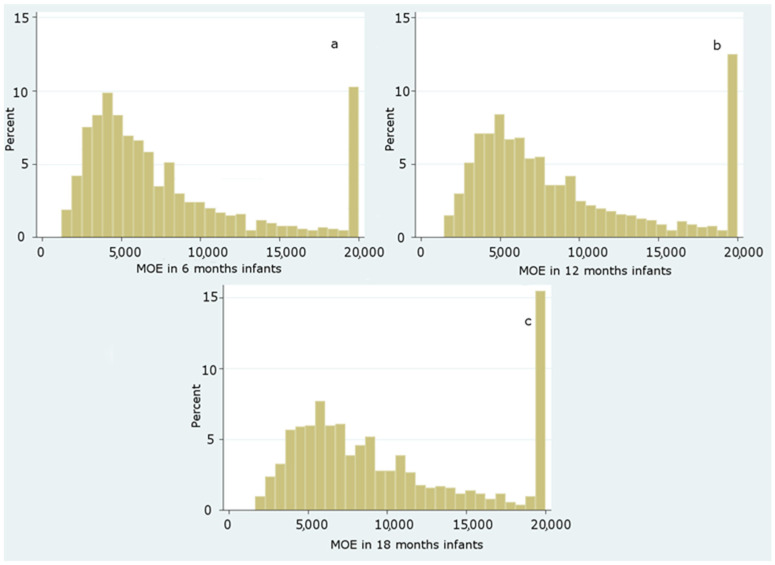
Distribution of MOE values for neurotoxic risk among infants at 6 (**a**), 12 (**b**) and 18 (**c**) months.

**Figure 3:**
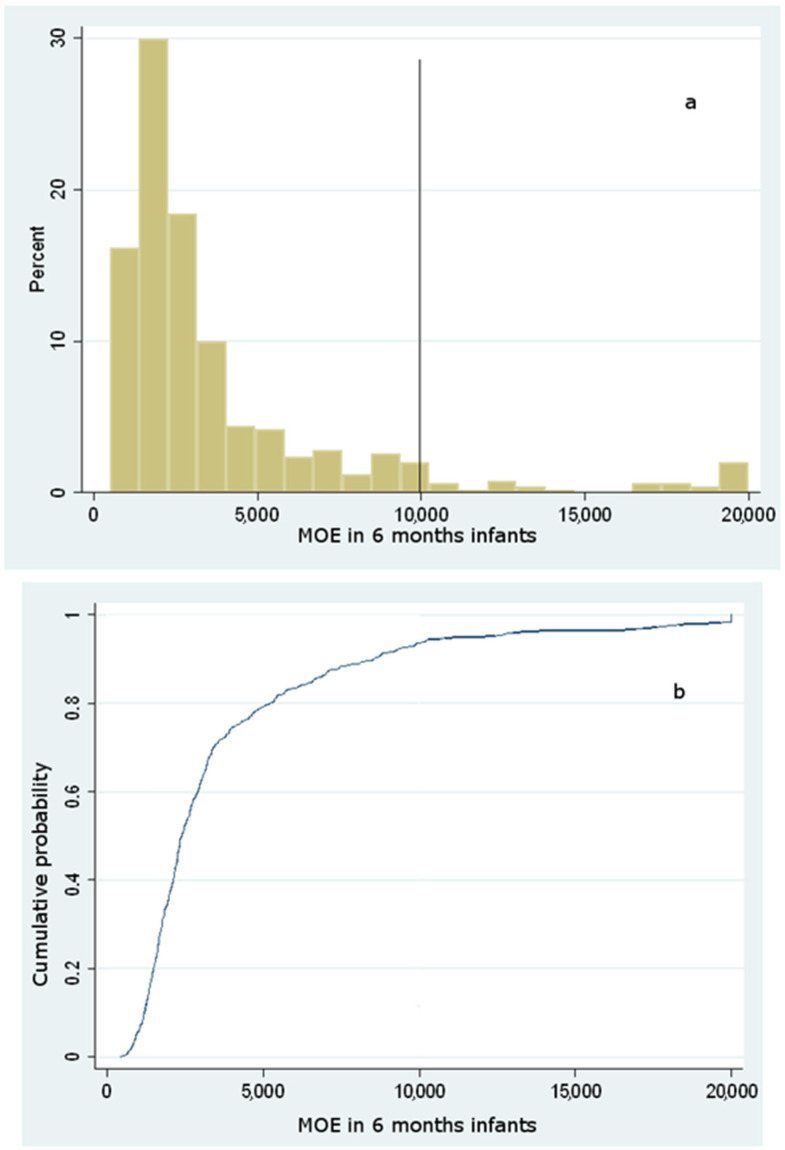
Histogram of simulated (**a**) and cumulative distribution function of simulated (**b**) MOE values for carcinogenic risk among 6 months infants considering a BMDL_10_ = 0.17 mg/kg bw/day.

**Figure 4:**
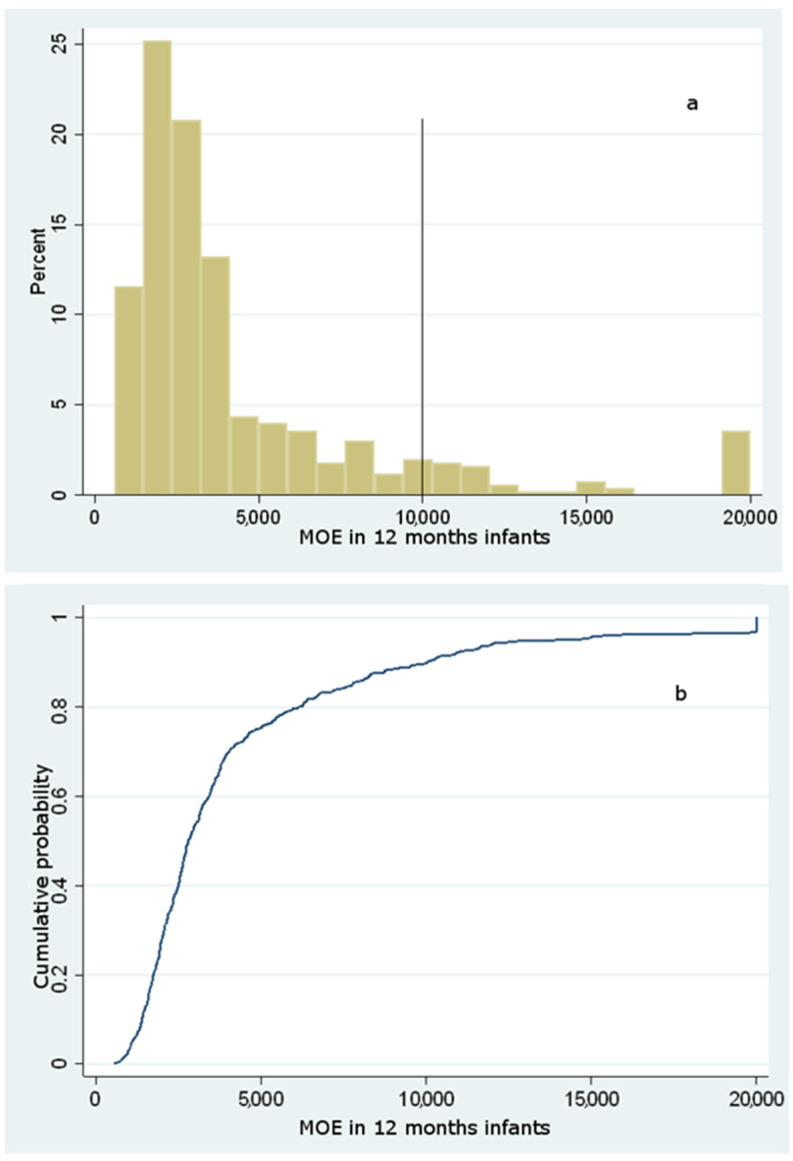
Histogram of simulated (**a**) and cumulative distribution function of simulated (**b**) MOE values for carcinogenic risk among 12 months infants considering a BMDL_10_ = 0.17 mg/kg bw/day.

**Figure 5:**
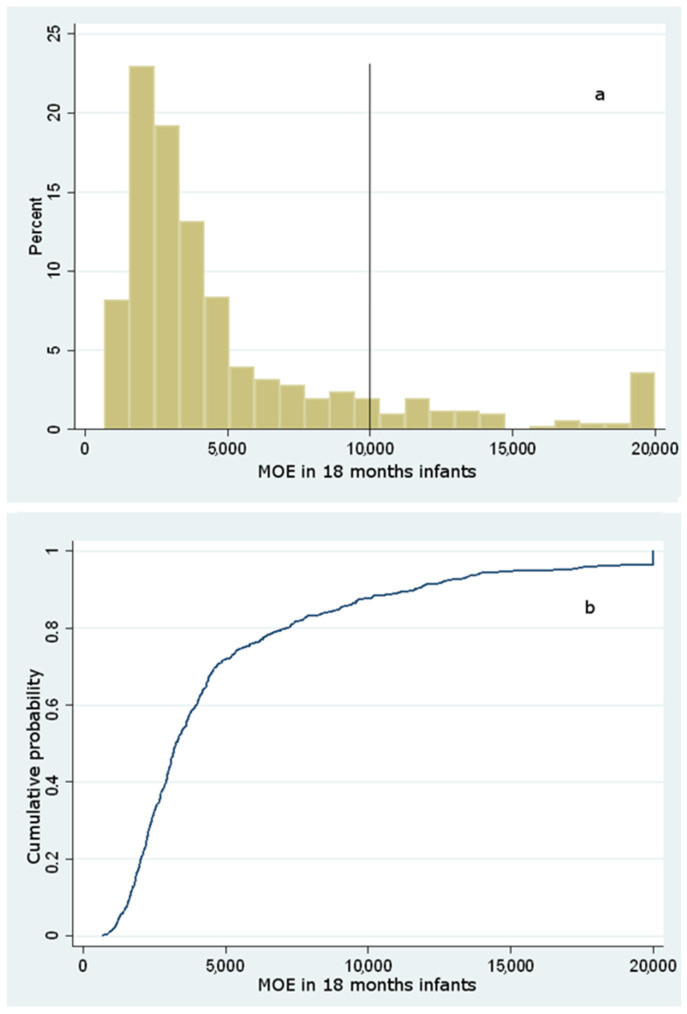
Histogram of simulated (**a**) and cumulative distribution function of simulated (**b**) MOE values for carcinogenic risk among 18 months infants considering a BMDL_10_ = 0.17 mg/kg bw/day.

Based on the data obtained, the distributions of MOE about neurotoxicity gave the following results (Figure 2):

A value of MOE greater than 125 is of low concern as regards the neurotoxic risk, whereas, regarding the carcinogenic risk, a MOE of low risk should be greater than 10,000 [21]. From the resulting data, 100% of the population studied showed a MOE well above 2000. Hence, no infant from 6 to 18 months shows neurotoxic concern. By contrast, two different carcinogenic endpoints were evaluated regarding the carcinogenic risk, considering two distinct BMDL_10_ values. Based on the results, the distributions of MOE taking into account a BMDL_10_ = 0.17 mg/kg bw/day for carcinogenicity gave the results as displayed in Figure 3, Figure 4 and Figure 5.

The analysis of the cumulative distribution curves illustrates that the likelihood of a carcinogenic MOE below 10,000 is most probable, accounting for a cumulative probability of 94%, 92%, and 87%, respectively, for 6-, 12- and 18-months infants. However, since the BMDL_10_ used for this evaluation is based upon non-reproducible carcinogenic evidence in humans (Harderian gland adenocarcinoma), the MOE was also assessed according to a higher BMDL_10_, coming out from mammary gland carcinoma in rats. Considering this latter assessment, the probability of carcinogenic risk for 6-, 12-, and 18-months infants was still high (85%, 81%, and 77%), outlining a significant risk for most of the population under study despite a higher BMDL_10_ (data not shown). These findings mirror those by Elias et al. [45], who reported a dietary intake lying in the range 0.12–0.80 μg/kg bw (0.66–2.34 as regards consumers at the 95th percentile): according to this study that considered commercial baby food purchased in Estonia, the MOE varied from 185 to 620 for carcinogenic effects, and from 467 to 1569 for neurotoxic effects, suggesting the need to reduce AA exposure among Estonian infants.

Therefore, given its consistency with previous deterministic and probabilistic assessments, this study suggests the need to delay the introduction of biscuits in the diet of weaned infants. Other implications raised by this study include possible future revision of threshold limits intended for infants, as far as the European Regulation is concerned, even though it should be kept in mind that AA development is an unavoidable process.

## 4. Conclusions

Overall, biscuits were considered as the primary source of AA intake among weaned infants. Even though no sample showed AA levels above the threshold limits issued by Regulation UE 2017/2158, this study strengthens the idea that introducing certain foods in the diet of weaned infants might lead to a significant probability of carcinogenic exposure.

In this regard, it is advisable to reduce the intake of these foods, as they are not essential for nutritional purposes either for the growth or development of weaned infants that should follow a balanced and varied diet instead, including plenty of fruit and vegetables as well as simple cereals, diminishing the number of biscuits and baked products. The issue of a more restrictive threshold regarding baked products intended for infants, albeit advisable, does not seem to be an easily achievable goal since the formation of AA is, in principle, an unavoidable process. Hence, delaying the introduction of such foods in infants’ diet may help maintain the MOE as low as possible.

This study cannot be considered exhaustive and is not exempt from limitations: only the consumption of whole and ground biscuits group have been considered, without including in the analysis other sources of AA intake; the use of the log-normal distribution to represent food consumption data has been sometimes criticized; and, finally, it could be appropriate to evaluate the likelihood of a concerning exposure also for older children, as this study, due to the limited consumption data on these groups, did not consider this sub-population for which carcinogenic risk, through the consumption of baked products, could be crucial as well. As a matter of fact, up to now, no epidemiological study or meta-analysis was able to demonstrate a clear relation between AA intake and cancer in humans. Therefore, further research needs to examine more closely the links between AA intake and neoplastic outcomes, especially amongst highly exposed individuals, also considering other dietary sources of exposure.

## Figures and Tables

**Table 1:** Summary statistics of the concentration of acrylamide detected in the samples.

Sample	Acrylamide (ng/g)				
	Mean ± SD	Median	95th Percentile	Min	Max
Biscuits (*n =* 20)	61 ± 20	61	79	<LOQ	109
Ground biscuits (*n* = 20)	39 ± 11	36	63	<LOQ	55
Multigrain meal (*n =* 14)	<LOD	<LOD	<LOD	<LOD	<LOD
Sweet snacks (*n =* 12)	<LOQ	<LOQ	<LOQ	<LOD	<LOQ
Savory snacks (*n* = 12)	<LOQ	<LOQ	<LOQ	<LOQ	<LOQ
Plum puree (*n =* 12)	<LOQ	<LOQ	32	<LOD	32

## Data Availability

Data and code are available upon request.
